# Cyclical Hemoptysis in a Patient With Right Lower Lobe Opacity

**DOI:** 10.1016/j.chpulm.2025.100215

**Published:** 2025-09-30

**Authors:** Shintaro Oyama, Tomonori Makiguchi, Yasuhito Nunomura, Shunta Mukai, Kengo Tani, Takahiro Sasaki, Daisuke Kimura, Masamichi Itoga, Hisashi Tanaka, Kageaki Taima, Sadatomo Tasaka

**Affiliations:** a Department of Respiratory Medicine, Hirosaki University Graduate School of Medicine, Aomori, Japan; b Department of Thoracic and Cardiovascular Surgery, Hirosaki University Graduate School of Medicine, Aomori, Japan; c Department of Clinical Laboratory Medicine, Hirosaki University Graduate School of Medicine, Aomori, Japan

## Abstract

A 33-year-old woman, who had a 6 pack-year smoking history, presented with cyclical hemoptysis for 4 months. Although she was treated for suspected pneumonia due to the opacity in posterior basal segment of the right lower lobe (Fig 1A), her symptom recurred concurrently with menstruation. She had a history of an induced abortion and a spontaneous abortion in her late 20s.

On the first visit to our department, her chest radiograph was normal. For the follow-up examination, the family doctor had performed chest CT scans during menstruation and the midportion of the menstrual cycle (Figs 1A, 1B). The chest CT scan in our hospital was performed premenstruation (Fig 2A). The CT scan during menstruation revealed a ground-glass opacity localized in the right S10 (right posterior basal segment), which was suggestive of hemorrhage. CT scan during the midportion of the menstrual cycle and premenstruation appeared to show a dilated distal bronchus in the right S10. When traced from the caudal to the cranial direction, the thickened portion was connected to the pulmonary artery. Therefore, we concluded that the thickened portion represented the accompanying pulmonary artery (Figs 2B-2E). She also underwent bronchoscopy, which showed no abnormal mucosal findings in any bronchus. We did not perform bronchoalveolar lavage because the ground-glass opacity disappeared by the time of bronchoscopy and we assumed that it would be difficult to adequately collect the bronchial lavage fluid from the posterior basal segment. She was started on combined oral contraceptives containing ethinylestradiol and norethisterone as empirical therapy. Because her symptoms did not improve, she received partial lung resection of the area surrounding the tiny lesion in the right S10. Because it was difficult to identify the dilated distal bronchus, intraoperative CT scan-guided localization using a hook-wire system was performed before this operation. Histopathologic examination of the specimen revealed hemosiderin-laden macrophages throughout the alveoli (Fig 3), suggesting repeated hemorrhage. There was a dilated bronchus in the subpleural area, accompanied by a pulmonary artery, both of which corresponded with the CT findings. The bronchus was lined with gland-like structures. Immunohistochemistry showed the gland-like structures were positive for CD10, progesterone receptor, and estrogen receptor, suggesting ectopic endometriosis (Fig 4).

**Figure 1:**
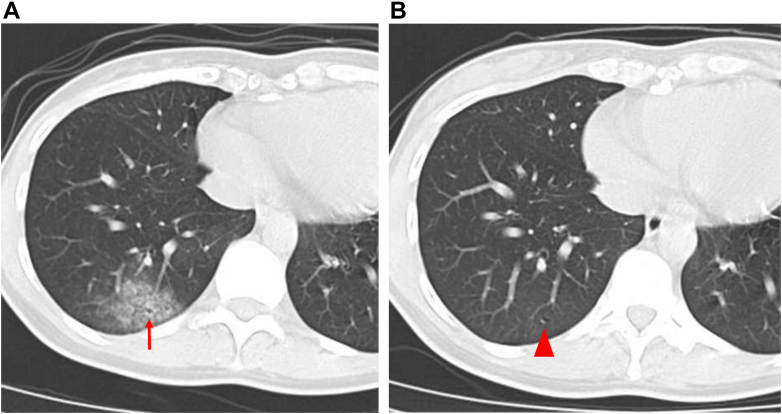
A, B, During menstruation and the midportion of the menstrual cycle CT images. A, CT scan conducted during menstruation demonstrates ground-glass opacity in the right lower lobe surrounding a cystic lesion (arrow). B, CT scan during the midportion of the menstrual cycle shows no ground-glass opacity but the remained dilated distal bronchus (arrowhead).

**Figure 2:**
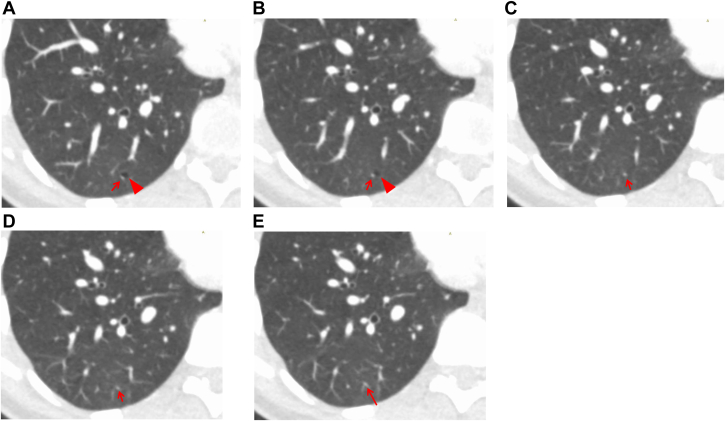
A-E, Successive premenstrual CT scans from the caudal to the cranial direction. Successive CT images lie from the caudal (A) to the cranial (E) direction. Arrows indicate pulmonary artery, and arrowheads indicate dilated bronchus.

**Figure 3:**
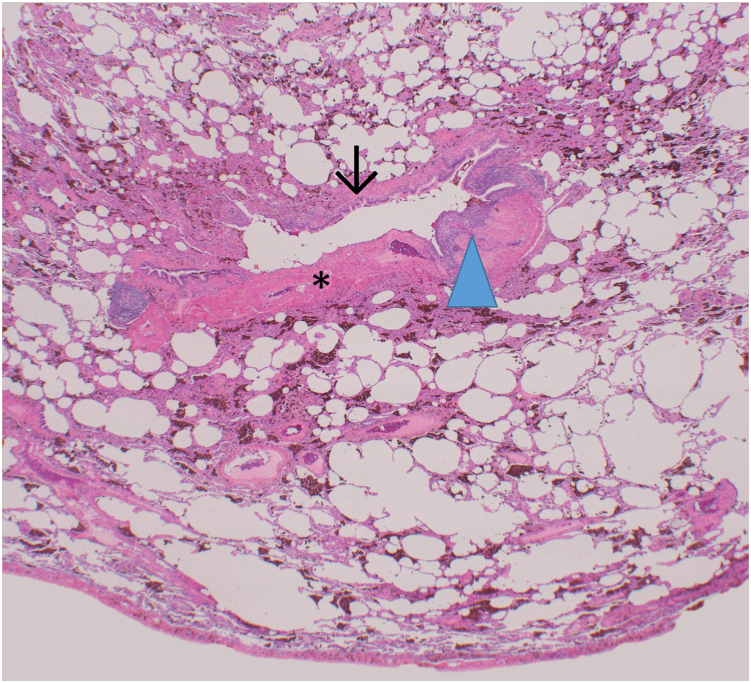
Pathologic examination of the resected right S10 lung specimen. Hematoxylin and eosin staining of the specimen with hemosiderin deposition and dilated bronchus (arrow), endometrioid-type stroma cells (arrowhead), and pulmonary artery (asterisk) (original magnification ×10).

**Figure 4:**
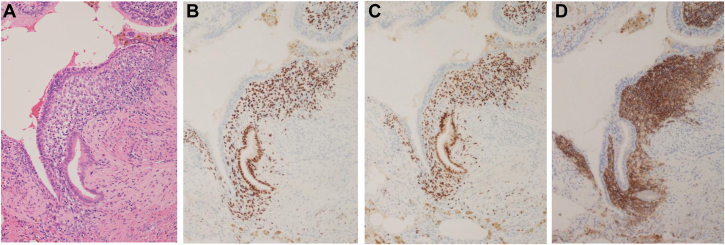
A-D, Immunohistochemistry staining of the specimen. A, Hematoxylin and eosin staining of endometrioid-type stroma cells (original magnification ×100). B-D, Immunohistochemistry staining for (B) progesterone receptors, (C) estrogen receptors, and (D) CD10 (original magnification ×100).

*What is the diagnosis?*

*Diagnosis:* Peripheral bronchial endometriosis

## Discussion

### Clinical Discussion

Endometriosis is characterized by growth of the endometrium outside the uterine cavity and is associated with infertility, dysmenorrhea, menorrhagia, and chronic pelvic pain. This condition affects women of reproductive age. Thoracic endometriosis is a rare form of ectopic endometriosis manifesting as pleural, parenchymal, or diaphragmatic endometriosis.^1^ Patients with thoracic endometriosis often experience a constellation of temporal symptoms such as catamenial pneumothorax, hemothorax, and hemoptysis.^1^ Kuo et al^2^ reported a case of submucosal bronchial endometriosis emerging only during menstruation, which was macroscopically identified and cytologically confirmed by bronchoscopy. To our knowledge, this is the first report demonstrating endometriosis in a peripheral bronchus.

Although the mechanism by which ectopic endometriosis develops is unknown, some theories exist. The most common hypothesized mechanisms, known as Sampson’s theory, is that when the menstrual blood flows backward, the shed endometrium can enter the pelvic cavity through the fallopian tube and implant outside the uterus.^1^^,^^3^ Local injuries to the cervix, vagina, and vulva can therefore easily cause endometriosis in the pelvic cavity.^3^ Peritoneal fluid containing endometrial cells goes through the right paracolic gutter to the right hemidiaphragm because of the falciform and phrenicocolic ligaments.^1^ Because of this, it is considered that right-sided thoracic endometriosis is typical. Patients with thoracic endometriosis present with pneumothorax, hemothorax, or hemoptysis. Although its diagnosis is based on clinical presentation and radiographic and histopathologic findings, many cases lack histopathologic confirmation to avoid invasive surgery. Recurrent hemoptysis is seen in various diseases (Table 1). Cardiovascular diseases may cause symptoms of heart failure, and infections present with fever and elevated serum inflammatory markers. Immunologic diseases are accompanied by systemic symptoms, such as joint symptoms and renal failure. Yao et al^3^ state that most patients with endometriosis of the lung had a history of miscarriage or uterine cavity surgery and typically present with repeated catamenial hemoptysis in previously reported cases. This suggests that uterine operations might facilitate the transfer of endometriosis cells to other organs, potentially via the bloodstream. On the other hand, there is no evidence that smoking is a risk factor for thoracic endometriosis. In this patient, a history of abortions might have contributed to the occurrence of thoracic endometriosis, whereas it was unclear whether the smoking history contributed to it. Regarding treatment for managing hemoptysis in thoracic endometriosis, hormone therapy is considered the first-line treatment. On treatment failure, surgery is an option.^1^ The patient presented with recurrent hemoptysis only during menstruation. Her symptoms resolved after surgery. She continued the combined oral contraceptives not to treat hemoptysis but to prevent menstruation. The reason why hormone therapy was not effective in this patient remains unknown. Previous studies have not described the detailed mechanism of hormone therapy on thoracic endometriosis. Flores et al^4^ showed that patients who did not respond to progestin-based therapies had significantly lower progesterone receptor levels than patients who did respond. Because combined oral contraceptives suppress luteal formation and maintain the endometrium at a constant level, it is possible that progesterone sensitivity was low in this case.

**Table 1:** Differential Diagnosis of Recurrent Hemoptysis

**Cardiovascular diseases**	**Neoplasm**	**Pulmonary parenchymal diseases**
Arteriovenous malformation	Bronchial adenoma	Diffuse alveolar damage
Pulmonary hypertension	Primary/metastatic lung cancer	Tuberous sclerosis
Pulmonary embolism/infarction	**Immunologic/vasculitic diseases**	Lymphangioleiomyomatosis
Mitral stenosis	Lupus pneumonitis	Pulmonary hemosiderosis
Thoracic aortic aneurysm rupture	Behçet disease/Hughes-Stovin syndrome	Interstitial fibrosis
**Infections**	Takayasu arteritis	**Others**
Lung abscess	Granulomatosis with polyangiitis	Bronchiectasis
Bronchitis	Goodpasture syndrome	Foreign body
Pneumonia	Antiphospholipid antibody syndrome	Dieulafoy disease of the bronchus
Fungal infection	IgA vasculitis	Systemic coagulopathy
Parasitic infection	Microscopic polyarteritis	Anticoagulants/thrombolytic agents
TBs/non-TB mycobacteria	Mixed cryoglobulinemia	Pulmonary endometriosis

(Adapted with permission from Larici et al.^5^)

### Radiologic Discussion

Radiologic findings in patients with thoracic endometriosis vary, including pneumothorax, pleural effusions, nodules, opacities, thin-walled cavities, segmental atelectasis, or bullae.^6^^,^^7^ Although these findings are nonspecific, chest CT scan continues to be the first-line modality for comprehensive evaluation and exclusion of other diseases. Using CT scan, we differentiated other causes of hemoptysis^5^(Table 1). Although lung infections, neoplasms, or some immunologic diseases may form nodules, they are generally thick-walled cavities and tend to grow over time. Arteriovenous malformations usually present as nodules without cavities. The nodule is accompanied by a feeding pulmonary artery and a draining vein. Some cystic lung diseases may be accompanied by thin-walled cysts, but they often present with diffuse and multiple cysts of various sizes. In this patient, the thickened portion in the right S10 was continuous with the more central pulmonary artery, and was identified as the accompanying vessel. Because a pulmonary artery is accompanied by a peripheral bronchus, the lesion is more likely to indicate a dilated peripheral bronchus rather than a cyst or another discontinuous lesion in the blood vessels. In pulmonary endometriosis, the lesion can show varying size and morphology over the menstrual cycle, or their disappearance between menstruations.^7^ Cyclic endometrial shedding in the lungs causes destruction of the lining of the alveolar epithelial cells, which is thought to lead to the formation of cysts and bullae.^8^ To date, there have been no reports regarding the radiologic features of peripheral bronchial endometriosis. We assume that the repeated hemorrhage and inflammation might have caused the localized bronchiectasis in this patient. MRI scan is a good option for the characterization of pleural endometriotic nodules and hemorrhagic pleural effusion.^9^ In this patient, the lesion showed no high signal intensity on T1- or T2-weighted MRI images.

### Pathologic Discussion

Thoracic endometriosis is histologically diagnosed by the presence of endometrial glands and stroma, with the stromal cells being positive for CD10, estrogen receptor, and progesterone receptor, markers suggestive of endometrial stroma.^10^ In pulmonary endometriosis, rupture of the capillaries or alveoli within the lesion during menstruation might result in hemoptysis or pneumothorax. To our knowledge, there have been no reports demonstrating a direct relationship between endometrial tissue and a peripheral bronchus. In this patient’s lung specimen, a mildly dilated bronchus was noted in the subpleural area (Fig 3). The dilated bronchus near the visceral pleura was consistent with the dilated distal bronchus on the chest CT scan. Although the bleeding site was located far from the central bronchi, the endometrial structure coexisted with the airway, which caused the patient to cough up blood. The successful management of hemoptysis depends on the accurate identification of the bleeding site. Although there are no reports regarding direct link between hormones and dilated bronchus, prostaglandin is possible to be involved. Prostaglandin E2 (PGE2) was shown to inhibit macrophage phagocytic ability of endometriotic cells.^11^ In addition, PGE2 are also known to induce vascular endothelial growth factor. As such, PGE2 may make ectopic bronchial endometriotic cells immortal, and repeated bleeding in bronchus leads to dilated bronchus. However, it is a matter of speculation.

## Conclusion

In cases of cyclical hemoptysis, pulmonary endometriosis, including peripheral bronchial endometriosis, should be considered. When evaluating patients with hemoptysis, clinicians should pay close attention to the dilated bronchus in the peripheral. The presence of an adjacent pulmonary artery might help identify a dilated distal bronchus.

## Financial/Nonfinancial Disclosures

None declared.
